# Polyfunctional antibody signature defines protection in Andes hantavirus survival

**DOI:** 10.3389/fimmu.2026.1730584

**Published:** 2026-06-05

**Authors:** Felipe Bravo, Tybbysay P. Salinas, Camila Kossack, Jorge T. Gonzalez, Israel Giacaman, Claudio Quevedo, Sebastián Fuller, Farides Saavedra, Claudio Aguilera-Pérez, Felipe Aguilera, Raúl Riquelme, María Luisa Rioseco, Mario Calvo, Raymond A. Alvarez, Juan Miguel Pascale, Blas Armién, Maritza Navarrete, Jose Luis Garrido, Maria Ines Barria

**Affiliations:** 1 Departamento de Microbiología, Facultad de Ciencias Biológicas, Universidad de Concepción, Concepción, Chile; 2 Facultad de Medicina, Universidad San Sebastián, Puerto Montt, Chile; 3 Departamento de Investigación de Enfermedades Emergentes y Zoonóticas, Instituto Conmemorativo Gorgas de estudios de la Salud, Ciudad de Panamá, Panama; 4 Laboratorio de Biología Molecular, Hospital Base de Valdivia, Valdivia, Chile; 5 Facultad de Ciencias Biológicas, Departamento de Bioquímica y Biología Molecular, Universidad de Concepción, Concepción, Chile; 6 Hospital Puerto Montt Dr. Eduardo Schütz Schroeder, Puerto Montt, Chile; 7 Instituto de Medicina, Facultad de Medicina, Universidad Austral de Chile, Valdivia, Chile; 8 Ichor Biologics LLC, New York, NY, United States; 9 Sistema Nacional de Investigación, Secretaria Nacional de Ciencia y Tecnología, Ciudad de Panamá, Panama

**Keywords:** ANDV, Ab profiles, Ab-dependent NK cell activation, ADCD, neutralization, non-neutralizing Ab effector functions

## Abstract

**Background:**

Andes orthohantavirus (ANDV) is a deadly pathogen that causes hantavirus cardiopulmonary syndrome (HCPS), which is a severe disease characterized by respiratory failure and high mortality rates (~30–40%). While previous studies have shown that neutralizing antibodies have a critical role in survival, the contribution of the Fc-mediated effector functions remains unexplored.

**Methods:**

We performed a serological profiling of acute HCPS patients and survivors, analyzing antibody types, subtypes, and neutralization capacity targeting ANDV glycoproteins (Gn and GnGc). Fc-mediated effector functions, which are key to defining antibody-mediated correlates of protection, were analyzed using FcγR reporter signaling assays, antibody-dependent NK cell activation, and antibody-dependent complement deposition (ADCD).

**Results:**

Acute HCPS patients who developed moderate disease exhibited significantly higher IgG levels against the Gn glycoprotein and stronger Fc-mediated effector functions, including antibody-dependent NK cell activation and ADCD against glycoproteins, compared to the more severe cases. Surviving acute patients showed elevated IgG and IgM responses against Gn. In survivors, polyfunctional non-neutralizing IgG activities persisted for years after infection and were more pronounced against the GnGc complex than Gn alone.

**Conclusion:**

In summary, our study reveals the existence of functional diversity in the humoral immune response to ANDV glycoprotein during HCPS, which can be associated with protective responses and may contribute to a long-lasting immune response to restrict ANDV infection. These findings identify Fc effector functions as important correlates of protection and provide valuable insights for the design of next-generation vaccines and therapeutics against hantaviruses.

## 1 Introduction

Andes orthohantavirus (ANDV) belong to the order Bunyavirales, family Hantaviridae and is the primary etiologic agent of hantavirus cardiopulmonary syndrome (HCPS) in South America, a severe disease characterized by rapid respiratory failure and high mortality rates (~30–40%) (1, 2). Among New World hantaviruses, ANDV is unique in its documented capacity for human-to-human transmission, underscoring its potential public health threat as an emerging viral infection (3, 4). Moreover, no vaccines or virus-specific therapeutics are currently available, and hospitalized patients receive only supportive care. Therefore, defining the immune correlates of protection in humans is critically important for the design and development of novel vaccines and therapeutics.

Humoral immune responses play a central role in controlling New World hantavirus (NWH) infection (5). Clinical and experimental studies show that higher levels of virus-specific IgG early in disease are associated with improved outcomes (6). Studies of Sin Nombre virus (SNV), the causative agent of HCPS in North America, further support a protective role for antibody responses, with higher IgG titers observed in convalescent individuals (7). Patients with milder disease also exhibit higher neutralizing antibody titers compared to those with severe disease (8), reinforcing that neutralization is a key contributor to protective immunity. In animal models, passive transfer of hantavirus-specific antibodies confers protection against ANDV-induced HCPS (9–12) Altogether, these findings demonstrate that humoral immunity is a key mediator of protection against NWH-associated HCPS.

The primary targets of humoral immune responses are the viral glycoproteins (GP) Gn and Gc, which assemble into heteromeric complexes on the surface of virions and mediate host cell attachment and entry (13). These glycoproteins are the only viral proteins exposed extracellularly and are therefore central to antibody-mediated recognition and control of infection. In neutralization studies, antibodies targeting distinct epitopes on Gn and Gc exhibit diverse functional properties, including differences in neutralization potency, mechanism of action, and protective efficacy *in vivo*, highlighting the importance of targeting specificity in shaping humoral immunity to hantaviruses (9, 13). These findings demonstrate that epitope and subunit targeting contribute to qualitative differences in antibody responses.

Beyond neutralization, antibodies exert antiviral activity by engaging innate immune effector mechanisms through their Fc domains (14). Upon formation of antigen–antibody immune complexes, the Fc region interacts with Fcγ receptors (FcγRs) expressed on innate immune cells such as natural killer (NK) cells, monocytes, and macrophages, triggering receptor crosslinking, intracellular signaling, and cellular activation (15). These interactions drive diverse antiviral functions, including antibody-dependent cellular cytotoxicity (ADCC), antibody-dependent cellular phagocytosis (ADCP), and antibody-dependent complement deposition (ADCD) (14).

Although direct evidence in New World hantavirus infection remains limited, prior studies support the biological relevance of Fc-mediated antibody activity. In SNV-associated HCPS, virus-specific IgG responses have been reported to be enriched for IgG3, an Fc-active subclass with strong Fcγ receptor and complement engagement potential (16). In addition, monoclonal antibodies targeting ANDV glycoproteins that confer protection *in vivo* following passive transfer have been shown to engage Fcγ receptors and mediate ADCC *in vitro* (9). However, the relative contributions of neutralizing and non-neutralizing antibody functions were not quantified in these studies. Altogether, these findings support a model in which Fc-mediated effector functions may contribute to protective immunity in hantavirus infection.

Despite the established importance of humoral immunity in hantavirus infections (8, 11, 17), the extent to which Fc-mediated antibody functions influence clinical outcomes in HCPS remains poorly defined. Specifically, it is unclear whether Fc-mediated effector functions distinguish protective from non-protective humoral responses or whether these features associate with survival. Furthermore, the contribution of glycoprotein subunit targeting in the context of non-neutralizing functions remains unexplored. To address this, we performed an integrated serological analysis of ANDV-infected individuals from a well-defined HCPS cohort in southern Chile, profiling antigen-specific antibody isotypes and subclasses, as well as their capacity to engage Fcγ receptors, activate NK cells, mediate complement deposition, and neutralize viral infection. Additionally, we examined differences in effector function activation between GnGc and Gn targeting responses. We hypothesized that qualitative differences in antibody effector function, rather than neutralization potency alone, define protective immune responses in ANDV HCPS, and that subunit targeting modulates the efficiency of Fc effector functions. By systematically evaluating these features, our study defines antibody signatures associated with survival and provides mechanistic insight into the role of Fc-mediated immunity in controlling ANDV infection.

## 2 Materials and methods

### 2.1 Human subjects

Human samples were collected after signed informed consent in accordance with the approval of Institutional Review Board protocols by the participating institutions (CEC-SSLR Ord N°399). Hantavirus disease survivors (n = 34) were recruited from databases across four regions in southern Chile, and blood samples were collected. These samples were taken between a range from 3 months to 14 years after infection, and they were classified as moderate if they needed supplemental oxygen as treatment and severe if they required mechanical ventilation. Acute sera (n=14) were provided by the reference hantavirus diagnostic laboratory at Hospital Base de Valdivia, according to their sample availability from IgM seropositive samples obtained between 2016-2018, which were collected between 1- and 10-days post symptoms onset (CEC-SSLR Ord N°233). Anonymized data from acute individuals were obtained from the Chilean hantavirus notification formulary, including demographic and clinical severity data. Healthy donors (n=18) were used as experimental controls. Samples were processed as previously described (18) and data were handled in accordance with Institutional Review Board requirements to ensure confidentiality.

### 2.2 ANDV GnGc and Gn recombinant protein expression and purification

Plasmids encoding soluble ectodomains of Andes orthohantavirus (ANDV) glycoproteins GnGc and Gn (strain Chile-9717869, NCBI code NP_604472.1) were designed as previously described (19). Protein expression and purification were performed using methods established for SARS-CoV-2 antigen production (20). ANDV GP were transiently expressed in Expi293F™ cells (Thermo Fisher Scientific) cultured in Expi293™ Expression Medium. Cells at 3 × 10^6^/mL were transfected using Polyethyleneimine (PEI MAX, Kyfora Bio) and incubated with transfection enhancers. After 72 h, supernatants were harvested by centrifugation and filtration. Clarified supernatants were diluted 1:1 with equilibration buffer (300 mM NaCl, 20 mM NaH_2_PO_4_·H_2_O, 10 mM imidazole, pH 7.4) and incubated with Ni-NTA HisPur™ resin (Thermo Fisher Scientific). Resin was washed with wash buffer (300 mM NaCl, 20 mM NaH_2_PO_4_·H_2_O, 25 mM imidazole, pH 7.4) and proteins were eluted with elution buffer (300 mM NaCl, 20 mM NaH_2_PO_4_·H_2_O, 250 mM imidazole, pH 7.4). Fractions were concentrated, dialyzed against 1X PBS, aliquoted, and stored at –20 °C.

### 2.3 ANDV specific ELISA

ELISA assays were performed in 96-well plates coated overnight at 4 °C with 2 μg/mL of ANDV GnGc or Gn recombinant proteins diluted in PBS. After coating, plates were washed with PBS containing 0.05% Tween-20 (PBS-T) and blocked for 1 hour at room temperature (RT) with PBS-T containing 5% non-fat dry milk. Heat-inactivated serum samples were then serially diluted three-fold and incubated for 1 hour at RT. After washing, plates were incubated with species-specific horseradish peroxidase (HRP)-conjugated secondary antibodies: goat anti-human IgM Fc5μ (1/3000 dilution), goat anti-human IgA α chain (1/2000 dilution), or goat anti-human IgG F(ab’)_2_ fragment (1/3000 dilution; all from Jackson ImmunoResearch). The reaction was developed with 3, 3’, 5, 5’-tetramethylbenzidine (TMB) substrate until visible color development and stopped with 2N sulfuric acid. Optical density (OD) was measured at 450 nm using a microplate reader. Antibody titers were calculated as the area under the curve (AUC) using GraphPad Prism.

For serum IgG subclass analysis, wells were incubated with serum samples at a 1/250 dilution for 2 hours at RT. Following three washes with PBS-T, wells were incubated with mouse anti-human subclass-specific monoclonal antibodies (ThermoFisher Scientific; all at 1/500 dilution): IgG1 (clone G17-1), IgG2 (clone G18-21), IgG3 (clone HP6050), or IgG4 (clone HP6025). After additional washes, bound antibodies were detected using HRP-conjugated goat anti-mouse IgG (H+L) secondary antibody (Invitrogen; 1/3000 dilution). After final washes, plates from survivor samples were developed with SIGMAFAST OPD substrate (Sigma-Aldrich), and the reaction was stopped with 3M HCl, OD was measured at 490 nm using a microplate reader. For acute samples, plates were developed with TMB substrate, and the reaction was stopped with 2 N sulfuric acid; OD was measured at 450 nm. Specific IgG subclass levels were calculated by subtracting the OD values of blank wells from antigen-coated wells.

For the detection of IgM antibodies against Andes virus nucleoprotein (NP-ANDV), 96-well plates were coated overnight at 4 °C with 0.3 μg/mL of recombinant NP-ANDV (provided by Dr. Maritza Navarrete, Molecular Biology Laboratory, Hantavirus Reference Laboratory, Hospital Base Valdivia). After washing with PBS-T, plates were blocked with PBS-T containing 5% non-fat dry milk for 1 hour at room temperature. Heat-inactivated serum samples were serially diluted three-fold and incubated for 1 hour at 37 °C. Following additional washes, bound antibodies were detected using HRP-conjugated goat anti-human IgM (Jackson ImmunoResearch; 1:3000 dilution in PBS-T) with 1 hour incubation at 37 °C. After final washes, the reaction was developed with TMB substrate, stopped with 2N sulfuric acid and OD was measured at 450 nm using a microplate reader. Antibody titers were calculated as AUC using GraphPad Prism.

### 2.4 Neutralization assay

Virus neutralization activity was quantified using a previously established pseudotyped particle neutralization assay (11). Pseudotyped particles were generated by co-transfecting HEK 293T cells with three plasmids: an envelope expression construct encoding the full-length ANDV glycoprotein precursor (GPC) from strain Chile-9717869, the HIV-1-based transfer vector pHR SIN CSGW encoding GFP and the packaging vector psPAX2. This design ensures presentation of conformationally intact GnGc epitopes that mimic those found on authentic ANDV virions. The pseudotyped particles bearing ANDV glycoproteins were harvested at 72 hours post-transfection, filtered through a 0.45-μm filter and stored at -80 °C for later use.

For neutralization assays, pseudotyped particles were pre-incubated with serial dilutions of heat-inactivated sera (1/50, 1/100, 1/500, 1/1000, 1/5000, 1/10000, and 1/20000) from ANDV acute, convalescent or healthy control subjects for 1 hour at 37 °C in 96 well culture plates. The serum-virus mixtures were used to transduce HEK 293 that expressed Integrin beta 3 cells (HEK 293T-IB3). After 16 hours of incubation, medium was replaced with DMEM supplemented with 10% CCS, L-glutamine, and penicillin-streptomycin. After 72 additional hours, cells were harvested using trypsin and resuspended in 0.5% paraformaldehyde in PBS. Cells were then analyzed for GFP expression by flow cytometry (BD LSRFortessa X-20). Data were acquired using FACSDiva software and analyzed with FlowJo (v10.8.1, Tree Star). The percent of virus neutralization was determined by calculating the normalized percent reduction in the number of GFP positive cells compared to control wells receiving only pseudotyped particles without serum.

### 2.5 Fc-gamma receptor signaling assay

To assess FcγRIIa and FcγRIIIa signaling, we employed our established reporter cell co-culture system (21), 293T cells were transfected with ANDV glycoprotein expression vector and 1.2x10^4^ transfected cells were preincubated with a 1/200 dilution of patient serum for 15 minutes at room temperature, then co-cultured for 16h at 37 °C with CD4+ Jurkat reporter cells (5:1 reporter:target ratio) expressing either FcγRIIa or FcγRIIIa along with an FcγR-responsive firefly luciferase reporter. After lysis (Promega E1531), IgG-dependent activation was quantified by measuring luciferase activity (Promega E1500) on a TECAN M200PRO plate reader, with background signals (from non-opsonized controls) subtracted to calculate specific activation in relative light units (RLUs). To assess FcγRIIb signaling, we generated a similar assay by modifying the surface receptor by fusing the extracellular domain of the receptor to the intracellular activating domain of FcγRIIa and generated a new reporter cell line with the same reporter gene. For FcγRIIb, identical experimental conditions were employed as those used for FcγRIIa and FcγRIIIa assessment. Sera from healthy hantavirus-seronegative donors (HD) were run in parallel for every cell line as the negative control group and are displayed in the graphs for comparison. All assays were performed in duplicates, and data are reported as raw relative light units (RLUs) on a log_2_ scale.

### 2.6 Antibody-mediated degranulation and activation of human NK cells

For the assay, high binding microplates (Corning) were coated with 4 μg/ml of ANDV GnGc or ANDV Gn at 4 °C overnight. Plates were blocked with 5% BSA, then incubated with serum diluted 1/100 in PBS for 2 hours at 37 °C. Unbound antibodies were removed by washing the wells 3 times with PBS prior to adding NK cells. Human NK cells were isolated from peripheral blood of human healthy donor’s buffy coats by negative selection kit (Miltenyi Biotec) the day before and rested overnight in 1ng/ml IL-15. The NK cells were added at 5 x 10^5^ cells/well in the presence of brefeldin A (BioLegend), Monensin (BioLegend), and anti-CD107a PE (BioLegend) and incubated for 5 hours at 37 °C. Following incubation, NK cells were surface stained with CD56 Alexa Fluor 647 (BioLegend) and subsequently fixed, permeabilized, and stained intracellularly for IFNγ Alexa Fluor 488 (BioLegend) and MIP-1β Brilliant Violet 421 (BioLegend) using the Fix & Perm cell permeabilization kit (Invitrogen) to detect the production of cytokine and chemokine. Control conditions included NK cells stimulated with antigen in the absence of serum to assess antibody-independent activation, as well as conditions lacking either NK cells or antigen to assess background signal. Samples were acquired on an Aurora CS spectral flow cytometer (Cytek), and data were analyzed using FlowJo software. The binding index (BI) was calculated by multiplying the percentage of CD56^+^ NK cells positive for CD107a, IFNγ, or MIP-1β by the median fluorescence intensity (MFI) of the corresponding signal, normalized to the MFI of healthy donor controls (18).

### 2.7 Antibody-dependent complement deposition

ANDV recombinant protein (ANDV GnGc or ANDV Gn) was covalently coupled to fluorescent carboxylate-modified beads (FluoSpheres Crimson 625/645 nm, F8816, Invitrogen) using a two-step carbodiimide reaction, as described earlier (22). Next, the complement deposition assay was performed as previously described, with modifications (23). Briefly, after overnight incubation, 7.6 × 10^6^ beads per tube were washed with PBS and incubated with 50 μl of diluted sera (1:10) from acute patients, survivors, and healthy donors for 2 hours at 37 °C. Following washes, Guinea Pig Complement Serum (Sigma), diluted 1:50 in RPMI-1640 (HyClone) supplemented with 10% FBS, was added and incubated for 15 minutes at RT. The beads were then washed and incubated for 15 minutes at RT with FITC-conjugated goat IgG specific to guinea pig complement C3 (MP Biomedicals) at a 1:100 dilution. As experimental controls, beads were processed in parallel with the omission of individual components, including (i) sera (to assess background complement deposition), (ii) Guinea Pig Complement (to confirm complement dependence), and (iii) FITC-conjugated anti-C3 antibody (to evaluate nonspecific fluorescence). BSA-conjugated beads were included as an additional control to assess antigen specificity and nonspecific antibody binding. Finally, the beads were washed and analyzed by flow cytometry using the Cytek Aurora CS. Events were gated on single beads, and positive bead events were identified in the R-1 detector for bead fluorescence. The assay readout included the median fluorescence intensity (MFI) and the percentage of FITC-positive beads detected in the B-2 channel. Data were expressed as a Binding Index, calculated by multiplying the percentage of C3-positive cells (FITC-positive beads) by the MFI of the signal, normalized to the average MFI of the negative controls.

### 2.8 Statistical analysis

Statistical and data analyses were performed using GraphPad Prism (version 10.4.1) and Python (version 3.13). The Mann–Whitney test was used to compare two groups. For comparisons among multiple groups, the Kruskal–Wallis test was applied, followed by Benjamini–Hochberg correction for multiple hypothesis testing. When significant differences were detected, *post hoc* pairwise comparisons were performed using a two-sided Mann–Whitney U test. Statistical significance was defined as p < 0.05 and is indicated as **** p < 0.0001; *** p < 0.001; ** p < 0.01; * p < 0.05.

For the heatmap analyses of humoral immune responses, antibody features were first log10-transformed and subsequently normalized using min–max scaling (0–1 range). Pairwise correlations between antibody titers and effector functions against ANDV GnGc or Gn antigens were calculated using Spearman rank correlation coefficient. Correlation matrices were visualized as heatmaps, where coefficients (r) are represented by color gradients ranging from blue (negative correlation) to red (positive correlation). Statistical significance thresholds were defined as **** p < 0.0001; *** p < 0.001; ** p < 0.01; * p < 0.05.

Polar bar plots were generated in Python using the pandas, numpy, and matplotlib libraries. For this analysis, acute patients were categorized into subgroups based on clinical characteristics and outcomes (disease severity and survival). To account for the non-normal distribution and wide range of values in the dataset, all numerical parameters were log10-transformed prior to normalization. Data were then scaled to the 0–1 range using min–max normalization across the full dataset to preserve inter-sample comparability. For each group, the mean normalized value for each marker was calculated. These mean values were then visualized using a polar bar chart to create a distinct profile for each patient group.

## 3 Results

### 3.1 Subject characteristics

A cross-sectional study was performed to characterize 14 acute ANDV HCPS patients (A01-A14), and 34 ANDV survivors (S01-S34). In addition, 18 healthy donors (HD01-HD18) were included as experimental controls. Clinical and demographic characteristics of the acute and survivor cohorts are summarized in **Table 1** and **Supplementary Table 1**.

**Table 1:** Clinical and epidemiological characteristics of the study population.

Cohorts	Acute (n=14)	Survivors (n=34)	Healthy donor (n=18)
Sex (n, %)			
Female	5 (36)	12 (35)	7 (39)
Male	9 (64)	22 (65)	11 (61)
Age (Years, mean ± SD)			
All	32 ± 13	46 ± 15	29 ± 5
Female	31 ± 17	48 ± 17	30 ± 6
Male	33 ± 12	45 ± 14	29 ± 5
Severity of HCPS (n, %)			
Moderate	6 (43)	12 (35)	NA
Severe*	8 (57)	22 (65)	NA
Time from disease onset (mean, range)**			
DDO	3.8 (1-10)	18869 (114-5371)	NA
YDO	<1	5.1 (0.3-14)	NA
Lethality of HCPS (n, %)			
Live	8 (57)	NA	NA
Dead	6 (43)	NA	NA
Risk factor for hantavirus infection			
Yes	NA	ND	7 (39)***
No	NA	ND	11 (61)

* Severe HCPS was defined as the requirement of mechanical ventilation at the time of hospitalization.

** Five acute patients and seven survivors with missing data were excluded from the analysis.

*** All risk factors are related with summer activities in rural areas such as camping, trekking, or visited uninhabited cabins at rural areas.

DDO, days from disease onset; YDO, years from disease onset, NA, not applicable; ND, not determined.

In the acute cohort, the mean age was 32 ± 13 years, and 9/14 (64%) were male. Eight of fourteen (57%) developed severe HCPS, defined by the requirement for mechanical ventilation at the time of hospitalization, and 6/14 (43%) died. Samples from this group were collected between 1- and 10-days post symptoms onset.

Among survivors (convalescent), the mean age was 46 ± 15 years, 22/34 (65%) were male. Twenty-two of thirty-four (65%) developed severe HCPS. Blood samples in this group were collected between 3 months and 14 years after disease onset, with all individuals considered fully recovered at the time of sampling. In the healthy donor’s group, the mean age was 29 ± 5 years, 11/18 (61%) were male. Eleven individuals (61%) reported no known HCPS risk factors such as camping, hiking, or visiting uninhabited cabins in rural areas (**Table 1**).

For acute-phase HCPS cases, diagnosis was confirmed by positive detection of anti-ANDV nucleoprotein (NP) IgM antibodies (all samples positive, **Supplementary Figure 1A**) (24).

### 3.2 Humoral immune profiles to ANDV Glycoprotein in acute and survivor HCPS subjects

Previous studies have reported increasing IgG titers against the ANDV GP during convalescence (6); however, the antigen-specific antibody isotypes and IgG subclasses remain poorly characterized. To address this gap, we first evaluated antibody responses directed against ANDV-GnGc and Gn in sera collected from acute phase patients and HCPS survivors.

**Figure 1:**
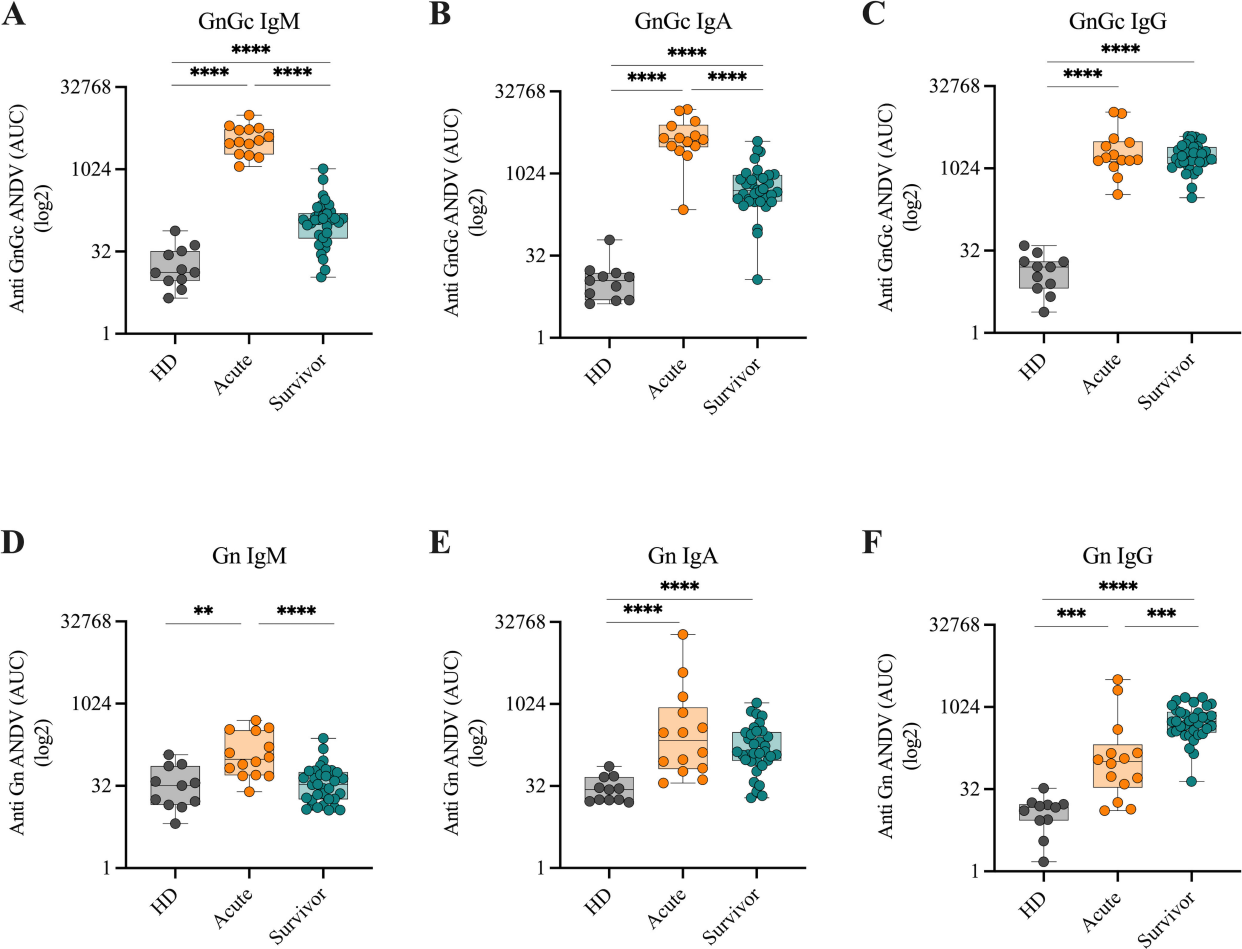
Differential ANDV GnGc and Gn-specific antibody responses in acute and survivor HCPS subjects. Serum levels of IgM, IgA, and IgG specific to recombinant ANDV glycoproteins were quantified by ELISA in acute HCPS patients (orange, n = 14), survivors (turquoise, n = 34), and healthy donors (HD, grey, n = 11). Results are expressed as area under the curve (AUC) values (log_2_ scale, Y-axis) and displayed as box plot. **(A–C)** Box plots show the titers of ANDV GnGc-specific antibodies and **(D–F)** Gn-specific antibodies. In each box plot, the central line represents the median, the boxes indicate the interquartile range (IQR), and whiskers denote the minimum and maximum values. Statistical analysis was performed using the Kruskal–Wallis test with Benjamini–Hochberg correction for multiple comparisons. Significant results were followed by two-sided Mann–Whitney U tests. Significance is indicated as p-values (****p < 0.0001, ***p < 0.001, **p < 0.01, *p < 0.05).

Acute-phase patients exhibited significantly elevated levels of IgM, IgA, and IgG against GnGc compared to healthy donors (**Figures 1A–C**). In contrast, IgM and IgA levels were lower in convalescent individuals relative to those in the acute phase (**Figures 1A, B**), consistent with the expected decline of early-phase antibody responses over time. Notably, anti-GnGc IgG titers in survivors persisted at levels comparable to acute-phase patients, indicating a sustained humoral immune response that is detectable even years after infection (**Figure 1C**; **Supplementary Figure 1B**).

Given that humoral immune responses evolve to recognize distinct viral epitopes, we also examined the antibody responses to the Gn glycoprotein (**Figures 1D–F**). Acute-phase patients showed significantly elevated IgM, IgA, and IgG responses against Gn compared to healthy donors (**Figures 1D–F**). In contrast to GnGc responses, anti-Gn IgG titers in convalescent subjects were significantly higher than in individuals in the acute phase (**Figure 1F**) and remained detectable for years after infection (**Supplementary Figure 1C**).

To further characterize the quality of the persistent antibody response, we profiled IgG subclass distribution. Mostly of patients in the acute cohort presented IgG1 and all survivors exhibited strong IgG1 responses that persisted years after infection (**Supplementary Figure 1D**). Interestingly, IgG3 was detected in three acute patients and IgG4 was only detectable in three individuals in the survivor cohort but at significantly lower levels than IgG1. In contrast, IgG2 and IgG3 levels were comparable between survivors and healthy controls.

Finally, we assessed the neutralization capacity of sera samples, which has been associated with survival and favorable clinical outcomes in hantavirus infections (5, 25, 26). Both acute and convalescent cohorts exhibited significantly higher neutralization levels than healthy donors (**Supplementary Figure 1E**).

### 3.3 FcγR-activating antibodies to viral glycoprotein persist in hantavirus survivors

While studies on hantavirus immunity have primarily focused on neutralizing antibodies, effective anti-viral protection may also involve non-neutralizing Fcγ receptor-mediated effector functions. Evidence from other viral infections, including HIV and influenza, demonstrates that FcγR-activating antibodies can contribute to protection independently of neutralization capacity (27–29). These observations underscore the need for more comprehensive profiling of antibody responses against ANDV, that incorporates the examination of non-neutralizing functions.

**Figure 2:**
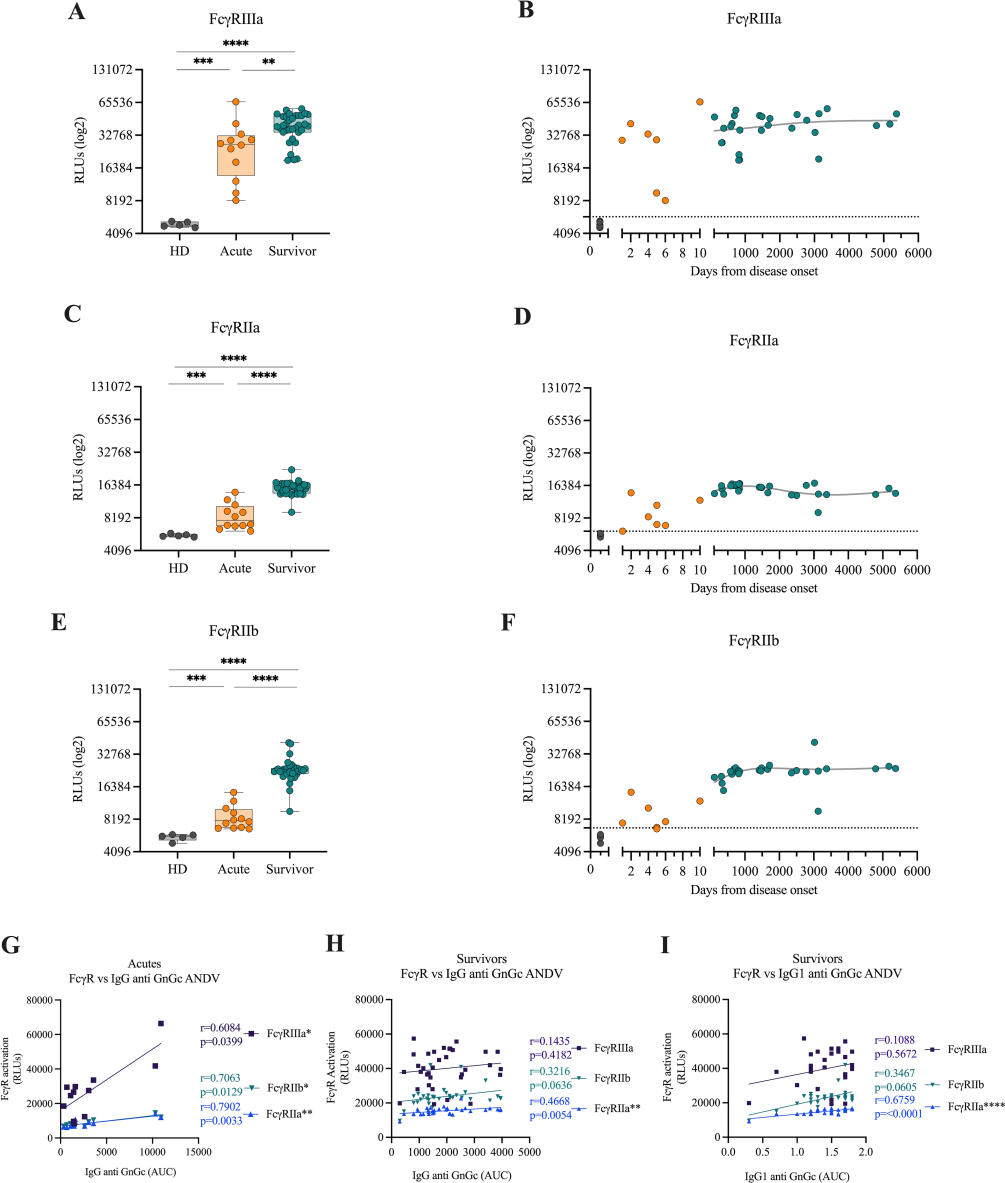
Differential activation profiles of Fc gamma receptors mediated by antibodies specific to ANDV glycoprotein in acute and survivors HCPS subjects. Activation of FcγRIIIa **(A, B)**, FcγRIIa **(C, D)**, and FcγRIIb **(E, F)** mediated by antibodies specific to ANDV glycoprotein was evaluated in sera from acute patients (orange, n = 12), survivors (turquoise, n = 34), and healthy donors (HD, gray, n = 5). Data are shown as relative light units (RLUs) on a log_2_ scale (Y-axis). **(A, C, E)** Box plots showing FcγR activation levels across groups. The central line represents the median, boxes indicate the interquartile range (IQR), and whiskers denote the minimum and maximum values. **(B, D, F)** Scatter plots representing the FcγR activation levels as a function of days from symptom onset. Only samples with available time-point data were included: acute patients (n = 7), survivors (n = 27), and HD (n = 5). The dashed line represents 3 standard deviations above the HD mean, and the grey line indicates a non-parametric spline regression model. Statistical analysis was performed using the Kruskal–Wallis test with Benjamini–Hochberg correction for multiple comparisons. Significant results were followed by two-sided Mann–Whitney U tests. Significance is reported as Mann–Whitney p-values. **(G–I)** Correlation analyses between antibody levels and FcγR activation. Graphs show correlations between ANDV GnGc-specific antibody levels (X-axis, AUC) and FcγR activation (Y-axis, RLUs). Analyses were performed for IgG levels in acute patients **(G)**, IgG levels in survivors **(H)**, and IgG1 levels in survivor **(I)** groups. Correlation coefficients (r) and *p* values were calculated using Spearman’s correlation test. Each dot represents an individual sample, and the lines represent linear regression models. Significance is indicated as p-values (****p < 0.0001, ***p < 0.001, **p < 0.01, *p < 0.05).

To evaluate FcγR activation profiles, we employed a Jurkat cell-based reporter system expressing FcγRIIa, FcγRIIIa, or FcγRIIb with an NFAT-driven luciferase readout. Survivors exhibited significantly higher ANDV GP-specific FcγR signaling compared to both acute-phase patients and healthy donors (**Figures 2A–F**). This enhanced signaling was observed across all three receptors, indicating broad and sustained Fc-mediated immune responses. Remarkably, this elevated FcγR activity persisted in survivors for more than 10 years post-infection, demonstrating the long-term durability of Fc effector functions against ANDV.

Acute phase patients displayed significantly lower levels of FcγRIIIa, FcγRIIa and FcγRIIb activation when compared to survivors (**Figures 2A, C, E**) although levels were higher than those in healthy donors. Moreover, a significant positive correlation was observed between anti-GnGc IgG levels in acute-phase and FcγRIIIa signaling (r=0.6084, p=0.0399), FcγRIIb signaling (r=0.7063, p=0.0129) and FcγRIIa signaling (r=0.7902, p=0.0033) (**Figure 2G**).

In the survivors, antibody levels correlated with Fcγ receptor activation. Anti-GnGc IgG titers showed moderate but significant correlation with FcγRIIa signaling (r=0.4668, p=0.0054; **Figure 2H**). Notably, IgG1 subclass antibodies exhibited a stronger correlation with FcγRIIa activation (r=0.6759, p< 0.0001; **Figure 2I**), suggesting that IgG1 may be a key driver of sustained FcγR signaling in long-term immunity. These findings highlight the importance of IgG1 subclass antibodies in maintaining Fc receptor-mediated protection against ANDV during the convalescent phase.

### 3.4 Antibody-mediated NK cell activation against ANDV glycoprotein differ in acute and survivor of HCPS

Given the robust anti-GnGc and anti-Gn antibodies and sustained neutralizing activity observed in acute and survivor’s sera (**Figure 1**; **Supplementary Figure S1**), we proceeded to explore whether these antibodies could trigger innate effector functions. To assess this, we employed a well-established degranulation assay (30) to evaluate NK cell activation. This assay measures CD107a surface expression and intracellular proinflammatory cytokine and chemokine production by NK cells, IFN-γ and MIP-1β respectively. To quantify NK activity, responses were expressed as a binding index (BI), which integrates both the frequency and median fluorescence intensity of responding cells.

**Figure 3:**
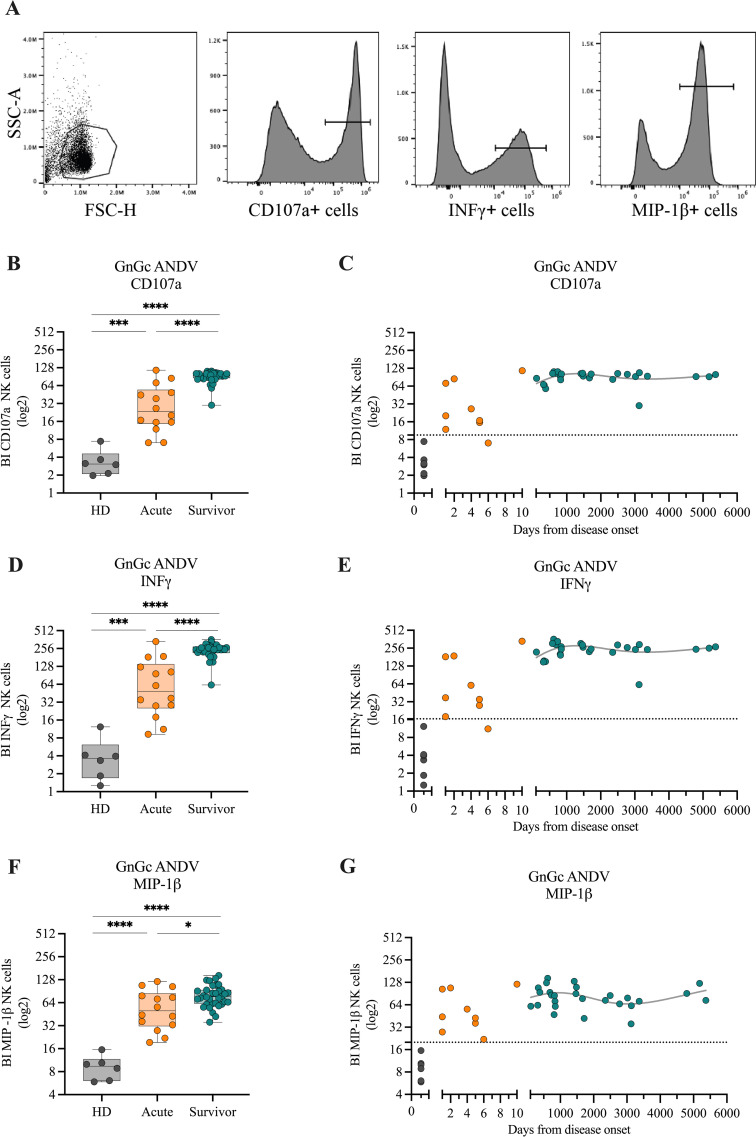
Antibody-dependent NK cell activation mediated by ANDV GnGc-specific antibodies in acute and survivors HCPS subjects. **(A)** Dot plot and histograms depicting the gating strategy for the antibody-dependent NK cell activation assay, CD107a +, IFNγ+, and MIP1β+ CD56+ NK cells from a representative survivor sample. **(B–G)** Antibody-dependent NK cell activation, expressed as binding index (BI) values (log_2_ scale, Y-axis), measured by the induction of CD107a **(B, C)**, IFN-γ **(D, E)**, and MIP-1β **(F, G)** expression in CD56+ NK cells upon incubation with immunocomplexes of ANDV GnGc with sera from acute patients (orange, n = 14), survivors (turquoise, n = 34), and healthy donors (HD, grey, n = 6). **(B, D, F)** Box plots showing NK cell activation across groups. The central line represents the median, boxes indicate the interquartile range (IQR), and whiskers denote the minimum and maximum values. **(C, E, G)** Scatter plots showing NK cell activation as a function of days from symptom onset. Only samples with available time-point data were included: acute patients (n = 9), survivors (n = 27), and HD (n = 6). The dashed line represents 3 standard deviations above the HD mean, and the grey line indicates a non-parametric spline regression model. Statistical analysis was performed using the Kruskal–Wallis test with Benjamini–Hochberg correction for multiple comparisons. Significant results were followed by two-sided Mann–Whitney U tests. Significance is indicated as p-values (****p < 0.0001, ***p < 0.001, **p < 0.01, *p < 0.05).

**Figure 4:**
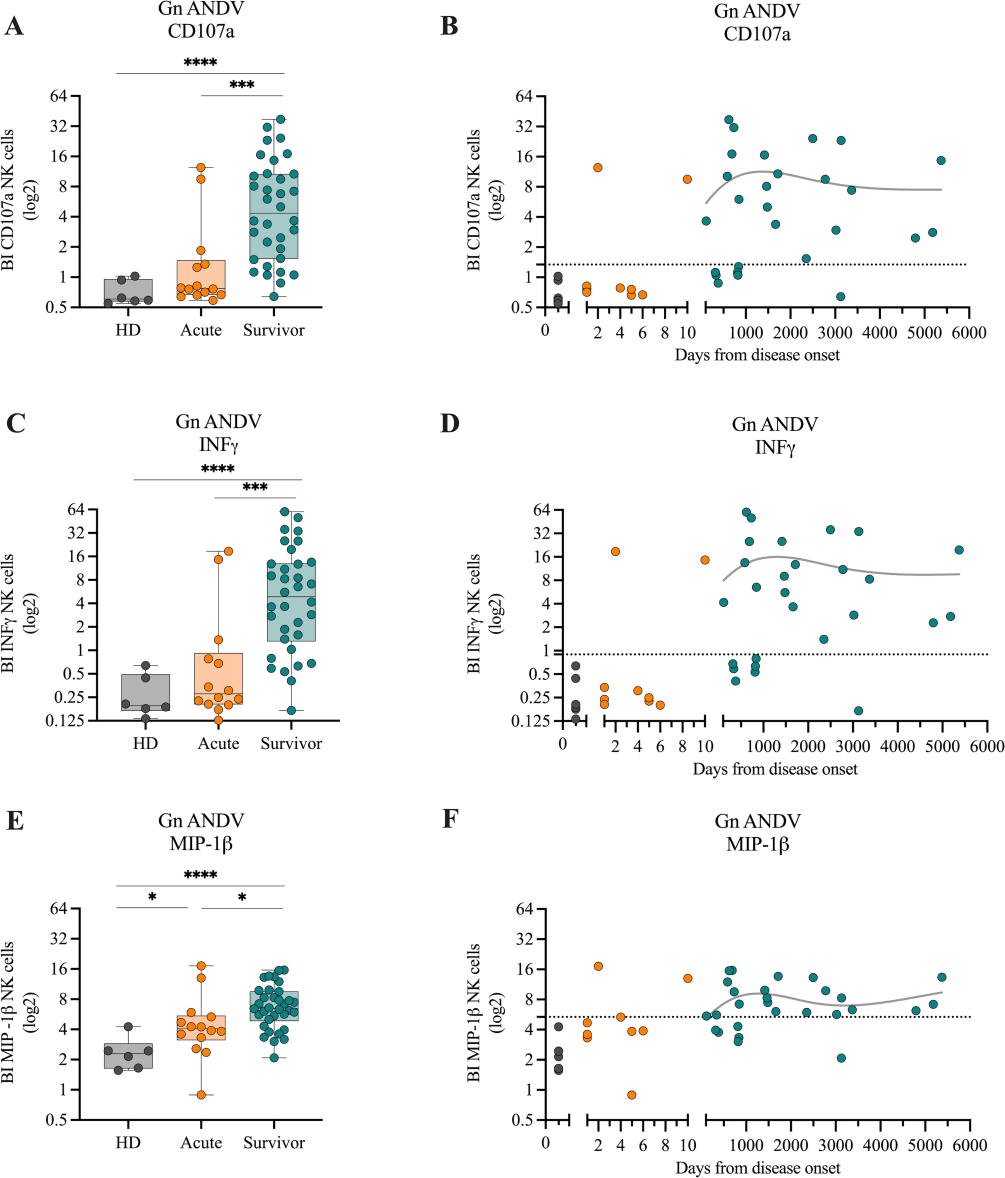
Antibody-dependent NK cell activation induced by ANDV Gn-specific antibodies in acute and survivor HCPS subjects. Antibody-dependent NK activation, expressed as binding index (BI) values (log_2_ scale, Y-axis), based on the induction of CD107a **(A, B)**, IFN-γ **(C, D)**, and MIP-1β **(E, F)** expression in CD56+ NK cells upon incubation with immunocomplexes of ANDV Gn with sera from acute ANDV patients (orange, n = 14), survivors (turquoise, n = 34), and healthy donors (HD, grey, n = 6). **(A, C, E)** Box plots showing NK cell activation across groups. The central line represents the median, boxes indicate the interquartile range (IQR), and whiskers denote the minimum and maximum values. **(B, D, F)** Scatter plots showing NK cell activation as a function of days from symptom onset. Only samples with available time-point data were included: acute patients (n = 9), survivors (n = 27), and HD (n = 6). The dashed line represents 3 standard deviations above the HD mean, and the grey line indicates a non-parametric spline regression model. Statistical analysis was performed using the Kruskal–Wallis test with Benjamini–Hochberg correction for multiple comparisons. Significant results were followed by two-sided Mann–Whitney U tests. Significance is indicated as p-values (****p < 0.0001, ***p < 0.001, **p < 0.01, *p < 0.05).

Antibody dependent NK cell activation revealed that GnGc antigen triggered greater NK cell degranulation (CD107a) in survivor’s sera compared to acute-phase patients and healthy donors (**Figures 3A–C**). This activation profile extended to cytokine and chemokine production, with survivor antibodies eliciting robust IFN-γ secretion (**Figures 3D, E**) and MIP-1β (**Figures 3F, G**) compared with acute-phase individuals and healthy donors. In contrast, the levels of antibody NK activation (CD107a, IFN-γ and MIP-1β) triggered by Gn antigen were lower as compared to antibody-dependent GnGc NK responses using survivor sera (**Figures 4A–F**), suggesting that antibodies directed to Gn are not the main driver of this effector function.

Acute-phase sera showed more variable NK cell activation. GnGc immune complexes induce higher antibody NK cell activation (CD107a, IFN-γ, and MIP-1β) compared with healthy donors but lower compared to survivors (**Figures 3B–G**). When Gn was used as the antigen, acute-phase antibodies induced significantly higher MIP-1β secretion as compared to healthy donors, but levels were lower than in survivor sera (**Figures 4A–F**).

Strikingly, GnGc-antibody complexes elicited the most robust overall NK cell responses, suggesting that antigen configuration may play a role in the efficiency of FcγR-mediated effector functions. Furthermore, the persistence of the GnGc-specific antibodies allowing NK cell activation in survivors, even years after infection, demonstrates the long-term maintenance of this response (**Figures 3C, E, G**).

### 3.5 Antibody-mediated effector functions against ANDV glycoprotein drive complement activation

**Figure 5:**
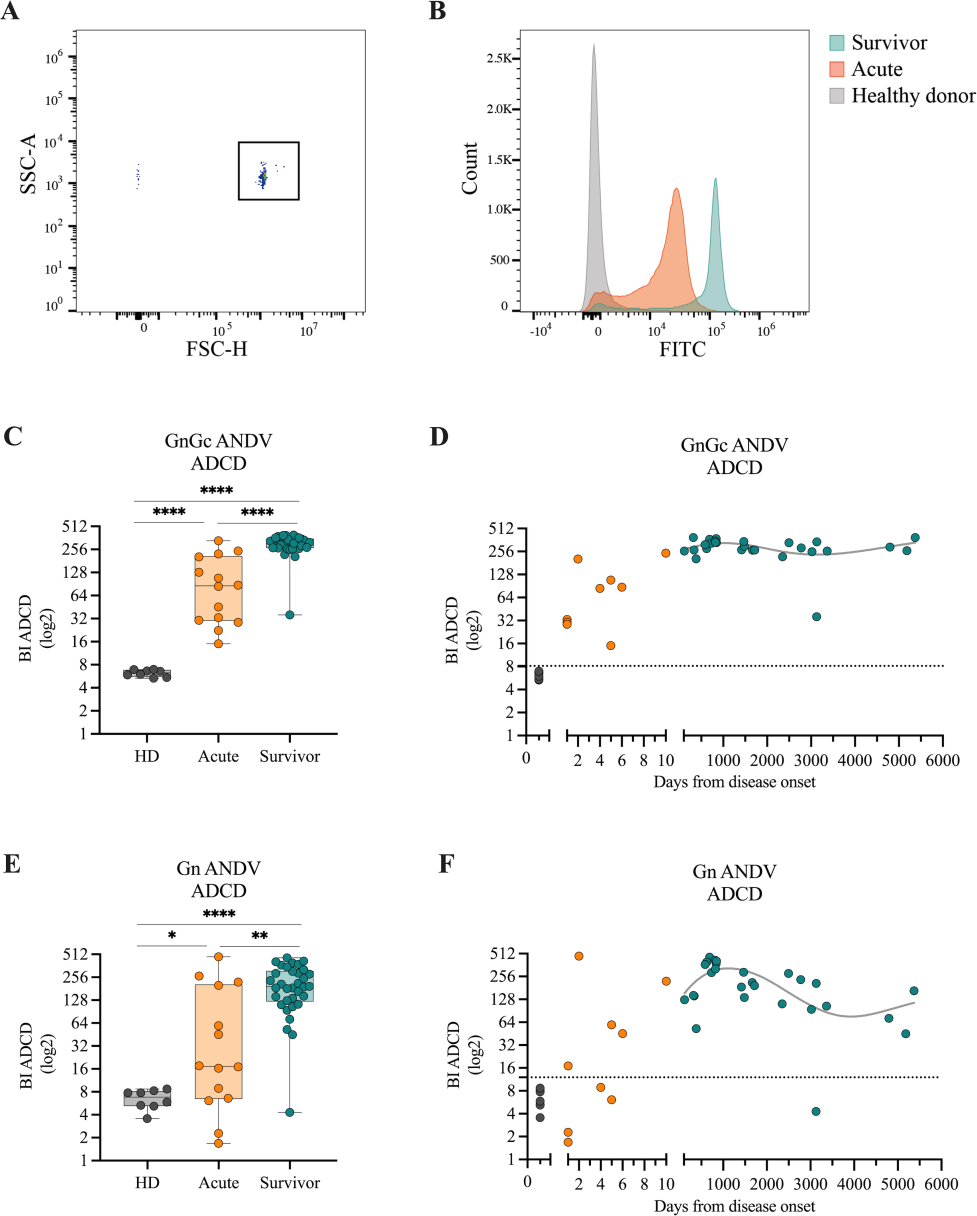
Complement-dependent activation associated with ANDV GnGc- and Gn- specific antibodies. **(A, B)** Gating strategy for the antibody dependent complement deposition (ADCD) assay by flow cytometry. **(A)** A dot plot depicts the gating strategy of Crimson positive beads and **(B)** histogram of FITC positive signal of a representative acute, survivor, and healthy donor samples. **(C–F)** ADCD activity, measured as complement binding index (BI) values, induced by ANDV GnGc-specific **(C, D)** and Gn-specific antibodies **(E, F)** in sera of acute ANDV patients (orange, n = 14), survivors (turquoise, n = 34), and healthy donors (HD, grey, n = 8). **(C, E)** Box plots showing ADCD activity across groups. The central line represents the median, boxes indicate the interquartile range (IQR), and whiskers denote the minimum and maximum values. **(D, F)** Scatter plots of ADCD activity as a function of days from symptom onset. Only samples with available time-point data were included: acute patients (n = 9), survivors (n = 27), and HD (n = 8). The dashed line represents 3 standard deviations above the HD mean, and the grey line indicates a non-parametric spline regression model. Statistical analysis was performed using the Kruskal–Wallis test with Benjamini–Hochberg correction for multiple comparisons. Significant results were followed by two-sided Mann–Whitney U tests. Significance is indicated as p-values (****p < 0.0001, ***p < 0.001, **p < 0.01, *p < 0.05).

To evaluate additional antibody effector mechanisms, we evaluated ANDV glycoprotein-specific complement activation using a quantitative ADCD assay. In this flow cytometry-based assay, microspheres coated with GnGc or Gn proteins were incubated with patient sera, followed by the addition of purified complement component C3. Complement activation was quantified by measuring C3 deposition on the antigen-coated beads as MFI (23) (**Figures 5A, B**).

We observed differences in the magnitude of ADCD among GnGc and Gn antigens, with higher levels of GnGc-specific antibodies. Survivor sera consistently triggered robust complement activation, against both GnGc (**Figures 5C, D**) and Gn antigens (**Figures 5E, F**), with significantly higher levels than acute-phase patients and the control group.

ADCD activity in acute-phase patients varied widely, we observed higher levels of antibody complement activation against GnGc than healthy donors (**Figures 5C, D**). However, although Gn-specific antibodies also elicited higher statistical ADCD levels compared to healthy donors, some acute-patient sera showed levels comparable to the control (**Figures 5E, F**).

These results establish that ANDV infection induces complement-activating antibodies during acute disease, with the most potent responses developing and persisting in survivors. These findings demonstrate that the induction of complement-activating antibodies can occur during acute disease and persist long after recovery. Together, these results demonstrate that complement-activating antibody responses are durable following ANDV infection and, alongside NK cell activation, reflect coordinated preservation of Fc-mediated immunity in survivors.

### 3.6 ANDV glycoprotein triggers polyfunctional antibody responses that differ between the acute and survival phases of HCPS

**Figure 6:**
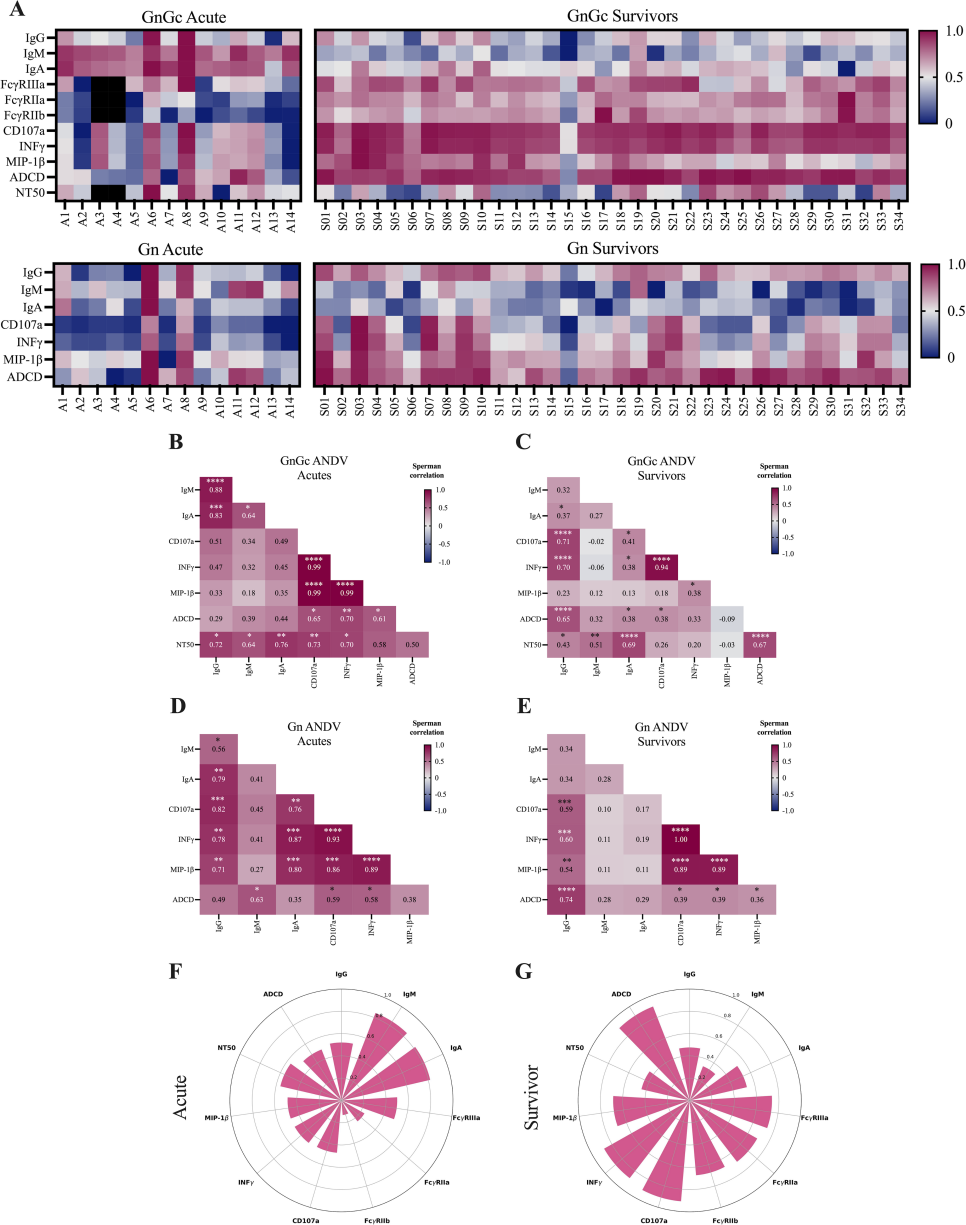
Dissecting differences in functional antibody responses between acute and survivor HCPS subjects. **(A)** Heatmap depicting antibody features against ANDV glycoproteins in acute patients (A01-A14) or survivors (S01-S34). Rows represents antibody features, including IgG, IgM, IgA, FcγRIIIa, FcγRIIa, FcγRIIb, CD107a, IFN-γ, MIP1β, ADCD, and NT50. All values were log_10_-transformed and subsequently normalized using min–max scaling. Responses are shown for the ANDV GnGc antigen (upper heatmap) and ANDV Gn antigen (lower heatmap). **(B–E)** Heatmaps showing pairwise Spearman correlation matrices between antibody titers and antibody effector functions against the ANDV GnGc **(B, C)** or ANDV Gn **(D, E)** in acute patients and survivors. Correlation coefficients (r) are displayed, with p scales of **** p < 0.0001; *** p < 0.001; ** p < 0.01; * p < 0.05. Positive correlations are shown in red, and negative correlations in blue. **(F, G)** Polar bar plots showing the mean of normalized values of antibody features targeting for ANDV GnGc (IgG, IgM, IgA, FcγRIIIa, FcγRIIa, FcγRIIb, CD107a, IFNγ, MIP-1β, and NT50) in acute patients **(F)** and survivors **(G)**. Bar length represents the mean of the normalized values for each antibody feature.

To comprehensively study antibody-driven functionality across disease stages, we generated a heat map integrating multiple antibody features, including antibody titers, FcγR activation, NK cell effector functions, ADCD, and neutralization capacity (NT50) (**Figure 6A**).

Overall, Fc-mediated effector functions targeting ANDV GnGc were more pronounced in survivors than in acute patients (**Figure 6A**). Notably, two acute-phase patients (A6 and A8) demonstrated exceptionally robust antibody effector activity specific to both ANDV GnGc and Gn, which was associated with moderate HCPS and favorable clinical outcome.

Despite heterogeneity among survivors, a similar functional antibody profile emerged for GnGc-specific responses amongst HCPS survivors (**Figure 6A**). Specifically, GnGc-specific antibodies elicited significantly stronger NK cell activation, as measured by CD107a expression, IFN-γ production, and MIP-1β secretion, as well as enhanced ADCD compared to Gn-specific responses (**Figure 6A**). This suggests a pattern of increased Fc receptor–dependent effector functions mediated by antibodies that recognize antigens beyond those exclusively targeting Gn during the convalescent phase.

To further define the relationship between antibody features, we performed correlation analyses using Spearman’s rank coefficients across both acute and survivor cohorts. In acute individuals, neutralizing activity (NT50) correlated significantly with GnGc-specific IgG (r=0.72, p=0.011), IgM (r=0.64, p=0.027) and IgA (r=0.76, p=0.006) titers (**Figure 6B**). In addition, NT50 showed significant positive correlations with NK cell markers CD107a (r=0.73, p=0.012) and IFN-γ p (r=0.70, p= 0.014) (**Figure 6B**). In survivor, correlations were broader and stronger (**Figure 6C**). Significant associations were observed between IgG and evaluated effector functions CD107a (r = 0.71, p= 0.001), IFN-γ (r = 0.70, p= 0.0008), ADCD (r = 0.65, p < 0.0001), as well as with NT50 (r = 0.43, p=0.010). Both IgM and IgA were also correlated with NT50 (**Figure 6C**).

Analysis of Gn-specific responses revealed a similar but more limited pattern. In acute-phase patients Gn-specific IgG correlated with CD107a (r = 0.82, p = 0.002), IFN-γ (r = 0.78, p = 0.0004), and MIP-1β (r = 0.71, p = 0.01) (**Figure 6D**). These correlations persisted in survivors, albeit at lower magnitude with IgG correlating with CD107a (r=0.59, p=0.0002), IFN-γ (r= 0.6, p=0.0002) and MIP-1β (r=0.54, p=0.03). ADCD also showed a strong correlation with IgG titers in survivors (r = 0.74, p < 0.0001) (**Figure 6E**). Overall, while Gn-specific antibodies were associated with Fc-mediated functions, these relationships were less extensive than those observed for GnGc. This indicates antigen-dependent differences in the breadth and strength of associations between antibody titers and non-neutralizing effector functions rather than the activation of entirely distinct effector mechanisms.

To further define global humoral response patterns, we generated polar plots interrogating all GnGc antibody features in the acute and survivor groups (**Figures 6F, G**). Acute patients were characterized by higher IgA and IgM titers and stronger neutralization activity. In contrast, survivors possessed higher antibody polyfunctionality, as exhibited by higher NK cell activity (CD107a, IFNγ, MIP1β), ADCD, and FcγRIIIa, FcγRIIa, FcγRIIb signaling (**Figure 6G**). Notably, despite comparable levels of GnGc-specific IgG between groups, effector function profiles were substantially enhanced in survivors.

Taken together, these findings suggest that ANDV infection induces distinct stage-dependent humoral immune profiles. While the acute phase is characterized by variable patterns depending on the specific viral antigen targeted by the antibodies, survivors developed robust, durable and polyfunctional antibody responses against the ANDV glycoprotein enriched for Fc-mediated effector functions that persist long after infection.

### 3.7 Humoral signature against ANDV glycoprotein is associated with disease severity in the acute cohort

**Figure 7:**
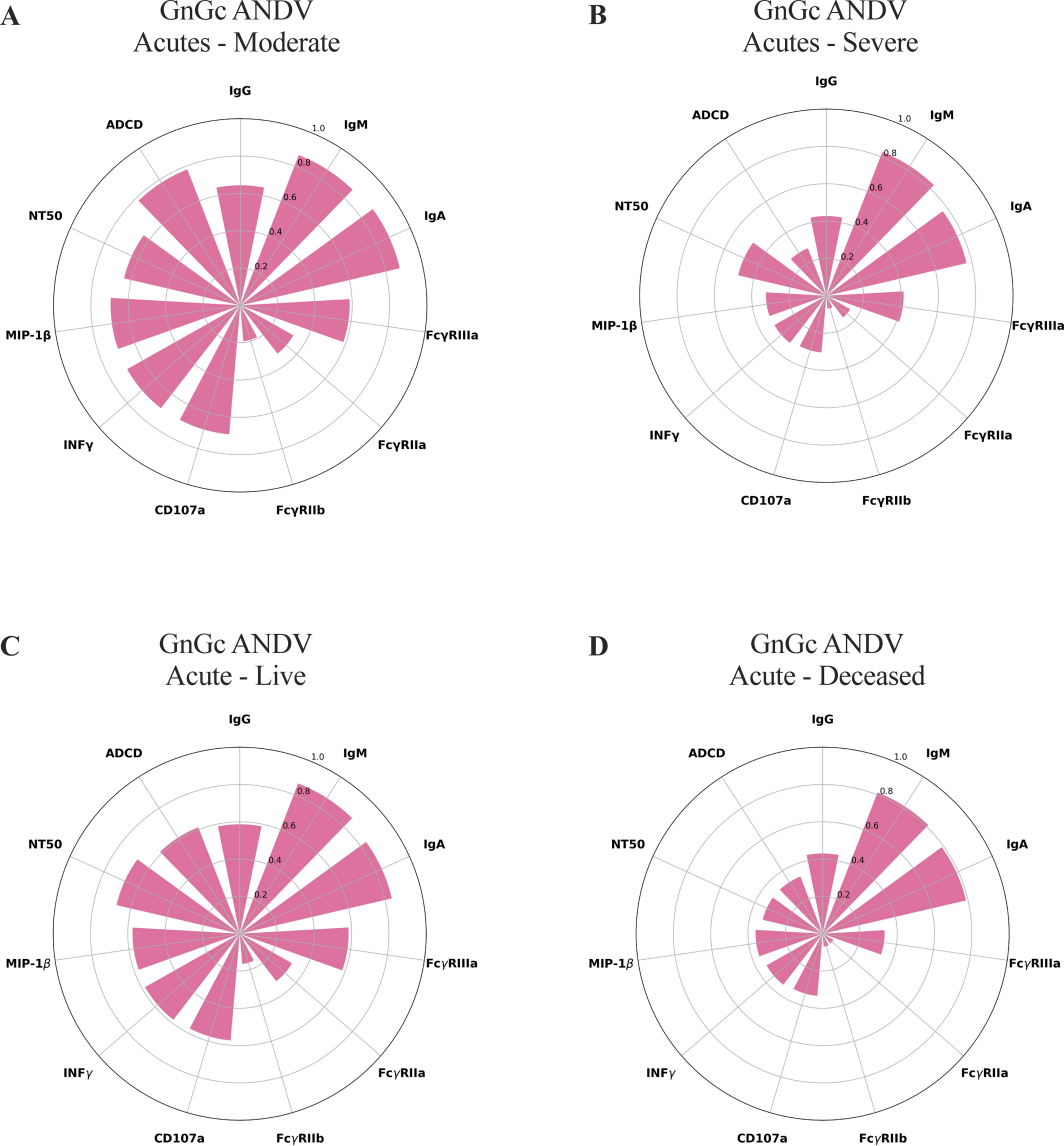
Polyfunctional antibody responses to ANDV GnGc are associated with disease severity in acute HCPS subjects. **(A, B)** Polar bar plots show the mean of normalized antibody feature values targeting GnGc (IgG, IgM, IgA, FcγRIIIa, FcγRIIa, FcγRIIb, CD107a, IFNγ, MIP-1β, and NT50) in the acute patient group, stratified by symptom severity as moderate **(A)** or severe **(B)**. **(C, D)** Polar bar plots show the mean normalized values for the same antibody features, stratified by clinical outcome as live **(C)** or deceased **(D)**. Bar length represents the mean of the normalized values of the corresponding antibody feature.

To investigate potential associations between immune parameters and clinical outcomes in ANDV infection, we compared immune response profiles during the acute phase between moderate and severe cases using box plots and polar plot analyses (**Figure 7**; **Supplementary Figures 2–4**).

Although not statistically significant, individuals with moderate disease (n=6) exhibited a trend towards higher GnGc (IgM, IgA and IgG) antibody titers as compared with severe individuals (n=8) (**Supplementary Figure 2A**). In contrast, anti-Gn IgG titers were significantly higher in moderate cases than in severe cases (**Supplementary Figure 2B**). This difference may reflect enhanced targeting of the Gn subunit, which is known to be more exposed and associated with receptor binding and neutralization (5).

Importantly, acute sera from patients with moderate disease elicited significant higher levels of all the Fc effector functions, including NK cell activation (CD107a, IFNγ, MIP1β) and ADCD against ANDV GnGc antigen (**Supplementary Figure 2A**). Similarly, antibody effector responses against Gn were also significant higher in moderate versus severe patients (**Supplementary Figure 2B**).

When we grouped the survivors into moderate or severe cases, no differences were observed in the antibody-mediated effector responses against either GnGc or Gn alone (**Supplementary Figure 3**). Although survivors maintained persistent polyfunctional antibody responses, the lack of differences likely reflects convergence of humoral immunity following recovery, limiting the ability to detect associations with initial disease severity.

To further identify the serological profiles associated with disease severity, we generated polar plots that incorporated all GnGc-specific antibody features from the acute-phase cohort (**Figure 7**). The analysis showed that while IgM and IgA levels were similar between the moderate and severe groups, moderate cases exhibited higher IgG levels, neutralization activity and enhanced Fc-mediated effector activity, suggesting more rapid or effective IgG class switching.

We next examined the humoral profile in acute-phase patients stratified by survival (**Supplementary Figure 4**). Individuals who survived exhibited a trend towards higher levels of anti-GnGc IgM, IgA, and IgG, as well as increased NK activation (CD107a, IFNγ, MIP1β) and ADCD compared to those who died, although these differences were not statistically significant (**Supplementary Figure 4A**). Similarly, for Gn-specific responses, surviving patients showed significantly higher IgM and IgG titers than deceased individuals (**Supplementary Figure 4B**), while other Fc-mediated features followed similar trends without statistical significance.

Polar plot analysis further highlighted these differences, revealing a more pronounced and polyfunctional antibody response in surviving acute-phase patients compared to those who died (**Figures 7C, D**). This profile was characterized by stronger IgG-associated effector functions and broader functional activity. Overall, these findings suggest that clinical outcomes are deeply influenced by both the quantitative and qualitative aspects of the humoral response.

## 4 Discussion

Our study reveals a clear functional antibody diversity in ANDV acute patients and survivors. In line with research on other pathogenic viruses, in which improved clinical outcomes depend not only on antibody quantity but also on the breadth of Fc-mediated effector activation, individuals who acquire moderate acute disease were associated with broad, polyfunctional antibody profiles (27, 31). On the other hand, a restricted response that is noticeably biased toward IgM and IgA isotypes was associated with poor outcomes (severe or fatal disease). This suggests ineffective maturation of the humoral response during acute infection, similar to patterns observed in fatal cases of other viral infections in which a lack of polyfunctional IgG responses is associated with increased mortality (31, 32). Conversely, we found that survivors exhibit a coordinated activation of diverse effector mechanisms including ADCD, antibody NK-cell activation (characterized by CD107a expression and production of MIP-1β and IFNγ), and neutralization (NT50). This polyfunctional antibody profile is maintained even years after recovery from infection, highlighting the durability of humoral immune imprinting following ANDV exposure. This long-term persistence underscores the potential relevance of polyfunctional antibody responses not only in acute protection but also in sustained immunity.

The hantavirus glycoproteins have been established as the principal targets of neutralizing antibodies (5). Studies in animal models and clinical observations consistently demonstrate that strong neutralizing antibody responses correlate with protection against hantavirus infection, including reduced disease severity in human cases of HCPS (7, 8). While non-neutralizing antibody functions have been recognized as critical correlates of protection in several viral infections, their role in ANDV immunity has remained unexplored. Notably, vaccine studies in the Syrian hamster ANDV model demonstrated that a single-dose adenoviral (Ad) vector platform expressing individual ANDV glycoproteins (Gn, Gc, or their combination) or nucleoprotein (NP) can confer sterile immunity against infection in the absence of neutralizing antibodies (33). These findings highlight the complexity of protective immunity against ANDV, suggesting that both neutralizing and non-neutralizing antibody functions may contribute to protection through distinct effector mechanisms.

The contribution of Fc-mediated effector functions mirrors clinical trials of vaccines against Ebola (34), influenza virus (35), HIV (36) and natural infection studies of Puumala virus (PUUV) (37), all of which have demonstrated that protection can be conferred by non-neutralizing antibodies through Fc-dependent effector mechanisms such as complement activation, antibody-dependent NK cell activation or phagocytosis. These mechanisms, acting in concert with the neutralizing response, may represent key components of protective immunity against ANDV where there has been evidence of a delayed T-cell response that is unable to resolve infection (17).

The predominance of IgG1 antibody response against the ANDV glycoprotein in acute and survivor HCPS patients, is consistent with other studies in patients infected with the European hantavirus strain PUUV (38) and the New World hantavirus CHOV (39). Although the 77% aminoacid identity (Clustal analysis) of the surface glycoprotein between SNV and ANDV, the IgG1 predominance contrasts with the reported IgG3 dominance in HCPS cases caused by the North American SNV strain (16), suggesting strain-specific differences in humoral immunity. Although both IgG1 and IgG3 are potent activators of ADCC and ADCP, IgG3 typically shows higher affinity for FcγRIIIa and is a more robust activator of the classical complement pathway (40, 41). This is in line with the profile observed in acute patients A6 and A8 that showed IgG3 positive seroconversion. This subclass divergence might have implications for the activation of FcγR-dependent effector functions in SNV; however, in our study, the strong NK activation and ADCD levels detected in acute and survivors demonstrate that IgG1 is highly effective in engaging these activities. On the other hand, IgG4 was only detected in three subjects of the survivor cohort suggesting potential class switching toward this IgG isotype. This finding is compatible with a previous report showing the presence of IgG4 in convalescent patients infected with PUUV, that could be associated to repeated or chronic exposure to antigen stimulation (37).

Additionally, both acute and survivor cohorts showed robust FcγR activation indicating a rapid and sustained Fc-effector functionality. This observation aligns with findings in a recent longitudinal study of PUUV infection (37) that show early detection of antibodies capable of activating FcγRIIIa. Interestingly, this study provides evidence of class-switching events with a shift toward IgG4 and a subsequent decline in effector function at months post-infection due to the lower affinity of this subclass to activating FcγRs. In contrast, our cross-sectional study provides a markedly different long-term immunological landscape for ANDV, as even years after infection sera from ANDV survivors contained antibodies capable of engaging both activatory FcγRIIa and FcγRIIIa, as well as the inhibitory FcγRIIb. Importantly, we found a strong correlation between antibody titers and FcγR activation in acute patients. However, in survivors, activation of FcγRIIIa and FcγRIIb receptors becomes independent of antibody levels. Accordingly, our results suggest that ANDV glycoprotein-specific antibodies from survivor’s experience affinity maturation rather than merely quantitative expansion, impacting immune activities mediated through FcγR engagement. Further studies are needed to confirm the underlying mechanisms.

We also found that elicitation of antibody-dependent NK cell activity against the glycoprotein GnGc was associated with moderate disease in acute patients. This finding is consistent with previous studies, which have shown that Fc effector functions are associated with improved clinical outcomes or reduced infection risk. In the HIV vaccine trial RV144, for example, where V1V2-specific IgG responses and ADCC activity were the primary correlates of reduced infection risk (42), with ADCC responses further linked to polyfunctional IgG3–IgG1 collaboration in protected vaccinees (43). Similarly, studies in Influenza, detected that HA-stem antibodies provide cross-strain protection primarily through ADCC-mediated clearance of infected cells (44). Fc effector functions have been shown to enhance the therapeutic activity of neutralizing monoclonal antibodies in animal models of SARS-CoV-2 (45). Even more, in SARS-CoV-2 infection, the functional properties of the antibody response have emerged as a key determinant of clinical outcome. While neutralizing antibodies are essential for preventing entry, studies have shown that survivors of severe COVID exhibit significantly higher ADCC activity compared to fatal cases, even when neutralization titers were comparable. Among patients with severe disease, survivors demonstrated greater ADCC activity compared to those who ultimately succumbed. Importantly, the detected ADCC was mediated by antibodies targeting different epitopes in SARS-CoV-2 (46). This is in line with our results, showing differences on antibody polyfunctionality that could be partially influenced by variations in antigen-specific targeting. Although moderate acute and live acute patients have significantly higher IgG titers against Gn, individuals with acute moderate disease and survivors had quantitatively and functionally superior responses against GnGc, characterized by elevated IgG levels, increased antibody Fc-mediated NK cell activation and ADCD. The enhanced immunostimulatory capacity of the full complex may be partly attributable to greater antibody abundance or could likely reflects the presentation of conserved, conformationally intact epitopes that optimize effector cell engagement, similar to the manner in which the more conserved HA stalk-binding antibodies confer protection primarily via Fc-dependent activation of immune effector cells including NK activation (47).

We also found that IgG antibodies are associated with ADCD and correlate with moderate disease in acute patients and survivors. This finding is consistent with studies on flavivirus infections, which have revealed that protection mediated by ADCD is associated with both the lysis of virions and virus-infected cells (48, 49). The complement system has been implicated in both protection and immunopathogenesis during hantavirus infections (50, 51). In PUUV acute phase patients, increased activation of the complement pathway has been observed in those with more severe clinical manifestations (52). In contrast, a study in patients infected with ANDV reported higher levels of complement factor C5/C5a in survivors compared to fatal cases (50), suggesting a potential protective role of the complement system in this context.

Interestingly, our study revealed comparable neutralization potency between cohorts, with significant titers of neutralizing antibodies even in the initial days following disease onset. Furthermore, the persistence of neutralizing antibodies over years is consistent with previous reports showing long-term maintenance of neutralizing antibodies after infection with ANDV, PUUV, and SNV (11, 37, 53, 54) indicating the induction of a long-lasting B cell memory response against hantaviruses. The detection of neutralizing antibodies in both acute and convalescent phases is consistent with prior studies showing plasmablast expansion early in hantavirus infection (55). The rapid emergence of neutralizing antibodies during acute infection and the early detection of IgA directed against the glycoprotein are probably explained by the massive CD27^hi^CD38^hi^ B cell response, which produces IgM, IgG, and IgA antibodies within days of symptom onset (55, 56). Notably, while this plasmablast wave is transient, the persistence of neutralizing antibodies in early and late convalescence phases (years after infection) suggest constant antigenic stimulation that allows the differentiation of a subset of activated B cells into long-lived plasma cells or memory B cell populations. In support of this idea, a follow-up study in a patient infected with ANDV reported the presence of viral RNA in a semen sample even seven years after the onset of symptoms (57) which is consistent with a possible restimulation of antiviral B cell responses due to viral persistence.

Altogether, our results propose that during ANDV infection there is a dynamic range of innate immune effector responses that are provided by the nature of the elicited antibodies, suggesting the existence of a coordinated participation of IgA- and IgG1-elicited antibodies that could neutralize viral particles and also be involved in ADCC and ADCD. Although we were not able to analyze ADCP function, this robust polyfunctional immune response could be associated with the absence of any reported symptomatic re-infections with any hantavirus (51), though definitive causal attribution is complicated by the sporadic nature of hantavirus exposure. This pattern of durable Fc-mediated immunity is consistent with observations in other viral infections. In SARS-CoV-2, neutralizing antibody titers decline more rapidly following natural infection than Fc-dependent effector functions, and this more durable non-neutralizing arm has been proposed to underpin residual protection against antigenically shifted variants such as Omicron (58).

Finally, one of the main questions raised by our study is whether the observed Fc-effector signatures are causative drivers of viral clearance or merely correlative markers of a robust immune response. As the present study is observational, we cannot definitively establish causality between polyfunctional Fc profiles and clinical outcome. Nevertheless, the strong association between broad Fc-mediated effector functions, ADCD and antibody-dependent NK cell activation and moderate disease in acute patients, together with the remarkable durability and evolution of these responses years after infection in survivors, suggests that Fc-mediated mechanisms may contribute to both acute viral control and long-term protection against ANDV. While neutralization titers remain an important correlate of protection, our data indicate that evaluating neutralization alone is insufficient to fully capture protective immunity. Thus, future efforts should focus on dissecting the contribution of Fc-mediated effector functions, as these are key for durable immunity following natural infection and should be considered as a correlation of protection in development of vaccine or therapeutic agents against ANDV.

## 5 Limitations of the study

This study has some limitations that should be acknowledged. First, the number of acute-phase HCPS patients included was relatively small reflecting the limited availability of samples from confirmed ANDV cases in Chile. Second, the sera scarcity limited our capacity to perform additional assays. Thus, this restricted sample size may reduce the statistical power to detect subtle associations, particularly when stratifying by disease severity or lethality. Third, the cross-sectional design of the acute cohort, in which samples were obtained at different days post-symptom onset, introduces heterogeneity that may influence the measured immune parameters. Similarly, for the survivor cohort, serum samples were collected across a wide temporal window ranging from months to more than a decade after infection, which may affect the comparability of immune responses within this group. Finally, as this was an observational study, causal relationships between specific antibody features and protection from severe disease cannot be definitively established. Despite these limitations, this study provides novel insights into the long-lasting polyfunctional antibody responses elicited by natural ANDV infection and underscores the importance of Fc-mediated effector functions in shaping protective immunity.

## Data Availability

The original contributions presented in the study are included in the article/**Supplementary Material**. Further inquiries can be directed to the corresponding authors.
