# Supplementary material for: Polyfunctional antibody signature defines protection in Andes hantavirus survival
Source: Front Immunol. 2026 Jun 5;17:1730584. doi: 10.3389/fimmu.2026.1730584 (PMC13279636; doi:10.3389/fimmu.2026.1730584)
Supplement: Supplementary file 1 [file SupplementaryFile1.docx]

Supplementary Material

**Table S1. Clinic and demographic characteristics of study subjects.**

| **Subject** | **Sex** | **Age** | **DDS** | **YDS** | **Group** | **Severity** | **Lethality** |
| --- | --- | --- | --- | --- | --- | --- | --- |
| A01 | M | 37 | 4 | 0,011 | Acute | Severe | Live |
| A02 | M | 59 | 6 | 0,0164 | Acute | Severe | Deceased |
| A03 | M | 22 | 1 | 0,0027 | Acute | Severe | Deceased |
| A04 | M | 25 | 1 | 0,0027 | Acute | Severe | Deceased |
| A05 | F | 27 | 1 | 0,0027 | Acute | Severe | Deceased |
| A06 | M | 25 | 2 | 0,0055 | Acute | Moderate | Live |
| A07 | F | 30 | 5 | 0,0137 | Acute | Severe | Live |
| A08 | M | 43 | 10 | 0,0274 | Acute | Moderate | Live |
| A09 | F | 58 | 5 | 0,0137 | Acute | Moderate | Live |
| A10 | M | 36 | NR | NR | Acute | Moderate | Deceased |
| A11 | M | 24 | NR | NR | Acute | Moderate | Live |
| A12 | M | 24 | NR | NR | Acute | Moderate | Live |
| A13 | F | 30 | NR | NR | Acute | Severe | Live |
| A14 | F | 12 | NR | NR | Acute | Severe | Deceased |
| S01 | M | 26 | 688 | 1,88 | Survivor | Moderate | Live |
| S02 | M | 56 | 336 | 0,92 | Survivor | Moderate | Live |
| S03 | M | 53 | 621 | 1,70 | Survivor | Moderate | Live |
| S04 | F | 34 | 1417 | 3,88 | Survivor | Severe | Live |
| S05 | M | 38 | 1483 | 4,06 | Survivor | Severe | Live |
| S06 | M | 27 | 383 | 1,21 | Survivor | Severe | Live |
| S07 | M | 51 | 733 | 2,16 | Survivor | Severe | Live |
| S08 | M | 38 | 1467 | 4,10 | Survivor | Severe | Live |
| S09 | F | 28 | 2497 | 6,92 | Survivor | Moderate | Live |
| S10 | M | 40 | 586 | 1,69 | Survivor | Moderate | Live |
| S11 | M | 43 | 4795 | 13,14 | Survivor | Severe | Live |
| S12 | F | 48 | 5181 | 14,19 | Survivor | Severe | Live |
| S13 | F | 41 | 1665 | 4,00 | Survivor | Severe | Live |
| S14 | M | 51 | 2351 | 6,44 | Survivor | Severe | Live |
| S15 | M | 58 | 3126 | 8,00 | Survivor | Moderate | Live |
| S16 | M | 68 | 114 | 0,31 | Survivor | Severe | Live |
| S17 | F | 68 | 3020 | 8,52 | Survivor | Moderate | Live |
| S18 | M | 48 | NR | NR | Survivor | Severe | Live |
| S19 | F | 78 | NR | NR | Survivor | Severe | Live |
| S20 | F | 29 | 5371 | 14,74 | Survivor | Moderate | Live |
| S21 | M | 54 | 3131 | 8,58 | Survivor | Severe | Live |
| S22 | M | 39 | 3369 | 9,00 | Survivor | Severe | Live |
| S23 | M | 68 | 839 | 2,00 | Survivor | Severe | Live |
| S24 | M | 26 | 814 | 2,23 | Survivor | Moderate | Live |
| S25 | M | 45 | 320 | 0,88 | Survivor | Severe | Live |
| S26 | M | 45 | 846 | 2,32 | Survivor | Severe | Live |
| S27 | F | 61 | 823 | 2,25 | Survivor | Severe | Live |
| S28 | F | 70 | NR | NR | Survivor | Severe | Live |
| S29 | F | 30 | 2778 | 7,00 | Survivor | Severe | Live |
| S30 | F | 46 | NR | NR | Survivor | Moderate | Live |
| S31 | M | 68 | NR | NR | Survivor | Moderate | Live |
| S32 | M | 26 | 1716 | 4,70 | Survivor | Moderate | Live |
| S33 | F | 42 | NR | NR | Survivor | Severe | Live |
| S34 | M | 31 | NR | NR | Survivor | Severe | Live |
| HD01 | F | 27 | NA | NA | Healthy donor | NA | Live |
| HD02 | F | 36 | NA | NA | Healthy donor | NA | Live |
| HD03 | M | 28 | NA | NA | Healthy donor | NA | Live |
| HD04 | M | 38 | NA | NA | Healthy donor | NA | Live |
| HD05 | M | 27 | NA | NA | Healthy donor | NA | Live |
| HD06 | F | 38 | NA | NA | Healthy donor | NA | Live |
| HD07 | F | 27 | NA | NA | Healthy donor | NA | Live |
| HD08 | M | 26 | NA | NA | Healthy donor | NA | Live |
| HD09 | M | 26 | NA | NA | Healthy donor | NA | Live |
| HD10 | M | 28 | NA | NA | Healthy donor | NA | Live |
| HD11 | M | 39 | NA | NA | Healthy donor | NA | Live |
| HD12 | M | 23 | NA | NA | Healthy donor | NA | Live |
| HD13 | M | 25 | NA | NA | Healthy donor | NA | Live |
| HD14 | M | 30 | NA | NA | Healthy donor | NA | Live |
| HD15 | F | 22 | NA | NA | Healthy donor | NA | Live |
| HD16 | M | 27 | NA | NA | Healthy donor | NA | Live |
| HD17 | F | 28 | NA | NA | Healthy donor | NA | Live |
| HD18 | F | 29 | NA | NA | Healthy donor | NA | Live |

DDO, days from disease onset; YDO, years from disease onset; NR, not reported; NA, not applicable.

# Supplementary Figures

**Supplementary Figure 1. Serum ANDV-specific antibody responses in acute and survivor HCPS subjects.**

**(A)** ANDV nucleoprotein (NP)-specific IgM antibody levels measured by ELISA in acute patients (blue, n=14) and healthy donors (HD, grey, n=12). Responses are presented as area under the curve (AUC) values (log₂ scale) and displayed as box plots. **(B and C)** Scatter plots show ANDV GnGc-specific (B) and Gn-specific (C) IgM, IgA, and IgG levels plotted over time since disease onset in acute patients, survivors, and HD. Only samples with available time-point data were included: acute patients (n = 9), survivors (n = 27), and HD (n = 12). The dashed line represents 3 standard deviations above the HD mean, and the grey line indicates a non-parametric spline regression model. **(D)** IgG subclass levels (IgG1, IgG2, IgG3, and IgG4) against ANDV glycoprotein (GP) measured by ELISA in acute patients (n= 14) and survivor group (n=30), levels are shown as optical density (OD) and displayed as box plots. **(E)** Neutralizing antibody responses against ANDV-GP pseudovirus in acute HCPS patients (orange, n = 12), survivors (turquoise, n = 34), and HD (gray, n = 13), expressed as IC50 values, data is displayed as box plots. In box plots the median is shown by the central line, boxes representing the interquartile range (IQR), and whiskers denoting the minimum and maximum values. Statistical analysis was performed using the Mann–Whitney U test for two-group comparisons. For multiple-group comparisons, the Kruskal–Wallis test with Benjamini–Hochberg correction was applied, followed by two-sided Mann–Whitney U tests for post hoc analyses. Significance is indicated as p-values (****p < 0.0001, ***p < 0.001, **p < 0.01, *p < 0.05).

**Supplementary Figure 2. Antibody features distinguish disease severity in the acute HCPS cohort.**

**(A-B)** Total IgM, IgA, and IgG antibody titers, NK cell activation markers (CD107a, IFN-γ, and MIP-1β), and ADCD activity directed against ANDV GnGc (A) or Gn (B) were evaluated in moderate and severe HCPS cases of the acute cohort (n = 6 and n = 8, respectively). Each dot represents an individual sample. Data are presented on a log₂ scale (y-axis) and displayed as box plots, where the median is indicated by the central line, boxes representing the interquartile range (IQR), and whiskers denoting the minimum and maximum values. Statistical comparisons were performed using the Mann–Whitney U test (p-value scale: ****p < 0.0001, ***p < 0.001, **p < 0.01, *p < 0.05).

**Supplementary Figure 3. Antibody features associated with disease severity in the survivor HCPS cohort.**

**(A-B)** Total IgM, IgA, and IgG antibody titers, NK cell activation markers (CD107a, IFN-γ, and MIP-1β), and ADCD activity directed against ANDV GnGc (A) or Gn (B) were evaluated in moderate and severe HCPS cases from the survivor cohort (n = 12 and n = 22, respectively). Each dot represents an individual sample. Data are presented on a log₂ scale (y-axis) and displayed as box plots, where the median is indicated by the central line, boxes representing the interquartile range (IQR), and whiskers denoting the minimum and maximum values. Statistical comparisons were performed using the Mann–Whitney U test (p-value scale: ****p < 0.0001, ***p < 0.001, **p < 0.01, *p < 0.05).

**Supplementary Figure 4. Association of antibody features with fatal outcomes in acute HCPS subjects.**

**(A-B)** Total IgM, IgA, and IgG antibody titers, NK cell activation markers (CD107a, IFN-γ, and MIP-1β), and ADCD activity directed against ANDV GnGc (A) or Gn (B) were evaluated in live and deceased HCPS cases from the acute cohort (n = 8 and n = 6, respectively). Each dot represents an individual sample. Data are presented on a log₂ scale (y-axis) and displayed as box plots, where the median is indicated by the central line, boxes representing the interquartile range (IQR), and whiskers denoting the minimum and maximum values. Statistical comparisons were performed using the Mann–Whitney U test (p-value scale: ****p < 0.0001, ***p < 0.001, **p < 0.01, *p < 0.05).
